# Musashi-1 Regulates MIF1-Mediated M2 Macrophage Polarization in Promoting Glioblastoma Progression

**DOI:** 10.3390/cancers13081799

**Published:** 2021-04-09

**Authors:** Yi-Ping Yang, Chian-Shiu Chien, Aliaksandr A. Yarmishyn, Man-Sheung Chan, Andy Chi-Lung Lee, Yi-Wei Chen, Pin-I Huang, Hsin-I Ma, Wen-Liang Lo, Yueh Chien, Wen-Chang Lin, Mong-Lien Wang, Ming-Teh Chen

**Affiliations:** 1 Division of Basic Research, Department of Medical Research, Taipei Veterans General Hospital, Taipei 112, Taiwan; ypyang3@vghtpe.gov.tw (Y.-P.Y.); cschien6688@gmail.com (C.-S.C.); yarmishyn@gmail.com (A.A.Y.); gghcy@gm.ym.edu.tw (M.-S.C.); cllee11@vghtpe.gov.tw (A.C.-L.L.); ychien@vghtpe.gov.tw (Y.C.); 2 School of Medicine, National Yang-Ming Chiao-Tung University, Taipei 112, Taiwan; chenyw@vghtpe.gov.tw (Y.-W.C.); pihuang@vghtpe.gov.tw (P.-I.H.); wllo@vghtpe.gov.tw (W.-L.L.); 3 Institute of Food Safety and Health Risk Assessment, School of Pharmaceutical Sciences, National Yang-Ming Chiao-Tung University, Taipei 112, Taiwan; 4 Institute of Pharmacology, National Yang-Ming Chiao-Tung University, Taipei 112, Taiwan; 5 Department of Neurosurgery, Taipei Veterans General Hospital, Taipei 112, Taiwan; 6 Cancer Center, Taipei Veterans General Hospital, Taipei 112, Taiwan; 7 Department of Neurological Surgery, Tri-Service General Hospital and National Defense Medical Center, Taipei 114, Taiwan; uf004693@mail2000.com.tw; 8 Division of Oral and Maxillofacial Surgery, Department of Stomatology, Taipei Veterans General Hospital, Taipei 112, Taiwan; 9 Institute of Biomedical Sciences, Academia Sinica, Taipei 115, Taiwan; wenlin@ibms.sinica.edu.tw; 10 Department of Medical Education, Taipei Veterans General Hospital, Taipei 112, Taiwan

**Keywords:** glioblastoma progression, M2 macrophages, macrophage inhibitory factor, Musashi-1, ISO-1

## Abstract

**Simple Summary:**

Glioblastoma (GBM) is the most lethal type of brain cancer. It is well known that the malignancy of cancers is dependent not only on the oncogenic properties of the tumor cells, but also on the composition of the tumor microenvironment, which includes macrophages of the immune system. The prevalence of M2 type macrophages usually promotes tumor progression as opposed to tumor-suppressing function of M1 type macrophages. In our previous studies, we identified Musashi-1 (MSI1) RNA-binding protein as a principal oncogenic factor in GBM. In this study, in a pursuit of finding secreted factors that may alter tumor microenvironment in GBM, we identified MIF1 cytokine to be positively regulated by MSI1. Moreover, we found that MSI1-mediated MIF1 secretion promotes differentiation of macrophages into pro-oncogenic M2 phenotype. The oncogenic role of MSI1/MIF1/M2 macrophage regulatory axis was also confirmed in GBM mouse models, which makes it a promising target for novel drug discovery.

**Abstract:**

Glioblastoma (GBM) is the most malignant brain tumor which is characterized by high proliferation and migration capacity. The poor survival rate has been attributed to limitations of the current standard therapies. The search for novel biological targets that can effectively hamper tumor progression remains extremely challenging. Previous studies indicated that tumor-associated macrophages (TAMs) are the abundant elements in the tumor microenvironment that are closely implicated in glioma progression and tumor pathogenesis. M2 type TAMs are immunosuppressive and promote GBM proliferation. RNA-binding protein Musashi-1 (MSI1) has recently been identified as a marker of neural stem/progenitor cells, and its high expression has been shown to correlate with the growth of GBM. Nevertheless, the relationship between MSI1 and TAMs in GBM is still unknown. Thus, in our present study, we aimed to investigate the molecular interplay between MSI1 and TAMs in contributing to GBM tumorigenesis. Our data revealed that the secretion of macrophage inhibitory factor 1 (MIF1) is significantly upregulated by MSI1 overexpression in vitro. Importantly, M2 surface markers of THP-1-derived macrophages were induced by recombinant MIF1 and reduced by using MIF1 inhibitor (S,R)-3-(4-hHydroxyphenyl)-4,5-dihydro-5-isoxazole acetic acid (ISO-1). Furthermore, GBM tumor model data suggested that the tumor growth, MIF1 expression and M2 macrophage population were significantly downregulated when MSI1 expression was silenced in vivo. Collectively, our findings identified a novel role of MSI1 in the secretion of MIF1 and the consequent polarization of macrophages into the M2 phenotype in promoting GBM tumor progression.

## 1. Introduction

Glioblastoma multiforme (GBM) is the most common and aggressive human brain cancer which is categorized as a grade IV tumor according to the World Health Organization [1]. GBM is classified as primary or secondary depending on the cause of formation. Primary GBM is malignant from the beginning and is highly prone to metastasis, whereas secondary GBM, which occurs in ~5% of glioblastoma patients, develops from lower-grade tumors [2]. Although surgical resection is the first-line therapy for GBM, adjuvant radiotherapy and chemotherapy are mandatory to achieve effective tumor control [3], such as by an alkylating agent temozolomide [4]. However, the outcomes of the treatment are limited, as most GBM tumors infiltrate deep into the brain tissues [5], which results in an average survival of only 12 to 15 months with less than 3–5% GBM patients surviving longer than five years [6].

Musashi-1 (MSI1) is a neural stem cell marker expressed at increased levels in a developing nervous system. This marker is also expressed abundantly in high-grade gliomas and correlates with poor prognosis. Evidence shows that MSI1 promotes tumor growth and radioresistance in both GBM and colon cancer [7,8]. In terms of RNA binding and regulation, MSI1 controls translation through binding with the specific motifs located in the 3′-UTRs of target mRNAs [9]. The most well-characterized mode of action of MSI1 is by inhibition of translation, in particular, its interaction with poly(A)-binding protein (PABP) was reported to disrupt the formation of an active translation complex [10]. However, MSI1 was also reported to promote translation, as was demonstrated in the case of ROBO3, a receptor involved in axonal guidance [11]. In gliomas, the expression of MSI1 was identified as a poor prognostic factor, whereby higher MSI1 expression was positively correlated with malignant progression of GBM, for instance, with cell proliferation, migration and angiogenesis [12,13].

In terms of microenvironment, GBM cells are characterized by heterogeneous immunogenicity which is the consequence of multifaceted immune composition that allows evading immune responses and eventually influences tumor progression and response to therapy [14]. Tumor microenvironment is heterogeneous and complicated and is formed by tumor and stromal cells, such as endothelial cells, pericytes, fibroblasts and extracellular matrix [15]. Among the hallmarks of immune response, immune infiltration by neutrophils, B lymphocytes, T lymphocyte subsets, myeloid-derived suppressor cells, natural killers (NKs), macrophages, and dendritic cells is a requirement for the initiation of a local immune response [16]. Among them, macrophages and neutrophils were found to adapt to the microenvironment and acquire different phenotypes [17]. Secreted factors or cytokines in the tumor microenvironment guide the macrophage development and functioning. For instance, some immunosuppressive factors like IL-10 and TGF beta can induce regulatory T cells to suppress cytotoxic T cell activation and blunt immune responses to attack cancer cells [15]. Accumulated evidence indicates that tumor microenvironment, especially its immune constituent, plays an important role in promoting GBM progression [18,19,20,21]. Recently, tumor-associated macrophages (TAMs), one of the types of infiltrating immune cells, were found to be abundant in GBM tissues and were proposed to play a key role in tumor growth [22]. Macrophages are known as immune antigen-presenting cells and the major type of the infiltrating immune cells contributing to inflammation, infection, and tissue damage [23]. Both innate and adaptive immune responses are governed by macrophages which also regulate tissue remodeling and repair [24,25]. However, apart from their protective function, there is increasing evidence that a subset of macrophages enhances pathogenesis and even tumor progression [25]. Macrophage polarization was defined by the distinct functions activated by different sets of cytokines or extracellular signals and resulted in either M1, the classically activated protective macrophages, or M2, the alternatively activated pathogenic macrophages [26,27]. In the tumor microenvironment, M2 TAMs are immunosuppressive and are induced by stimuli such as IL-4 and IL-13 [28,29]. Numerous studies revealed that M2 TAMs were activated and in turn promoted tumor progression under the guidance from tumor-released immunosuppressive cytokines and chemokines [1], while the breakdown of M2 TAMs effectively decreased GBM malignancy in an animal model [30]. Besides, polarizing macrophages from the M2 to the M1 phenotype was reported to result in anti-tumor immune responses and retard GBM tumor growth [31]. These studies indicate that M2 TAMs and the M1/M2 polarization mechanism in the tumor microenvironment are potential therapeutic targets for GBM treatment.

Interestingly, some studies linked MSI1 to macrophage activation and polarization, as it creates a tumor-friendly microenvironment by means of regulation of cytokine secretion and gene expression inhibition [32,33]. In a transcriptome-wide study, it was found that a homologous protein, Musashi-2 (MSI2), could affect IL-6 signaling [34], which echoes our previous finding that MSI1 activates Akt signaling and promotes IL-6 autocrine signaling [33]. Given that MSI1 is enriched in more malignant tumors while M2 TAMs are believed to be supportive in the progression of GBM, we hypothesized whether tumor microenvironment changes could be regulated by an MSI1-related mechanism. Here, we used THP-1 cells to monitor the activation and polarization of macrophages under modulation of MSI1 expression and then aimed to identify possible cytokines or secreted proteins using cytokine arrays. We identified macrophage inhibitory factor 1 (MIF1) as a cytokine regulated by MSI1 and mediating M2 polarization of macrophages, and such regulation was found to be associated with a malignant phenotype. These findings could be beneficial for establishing a potential therapeutic target to disrupt TAMs as well as for combating tumor-friendly microenvironment in GBM patients.

## 2. Results

### 2.1. Musashi-1 Overexpression in GBM Cells Elevates MIF1 Expression and Secretion

**Figure 1:**
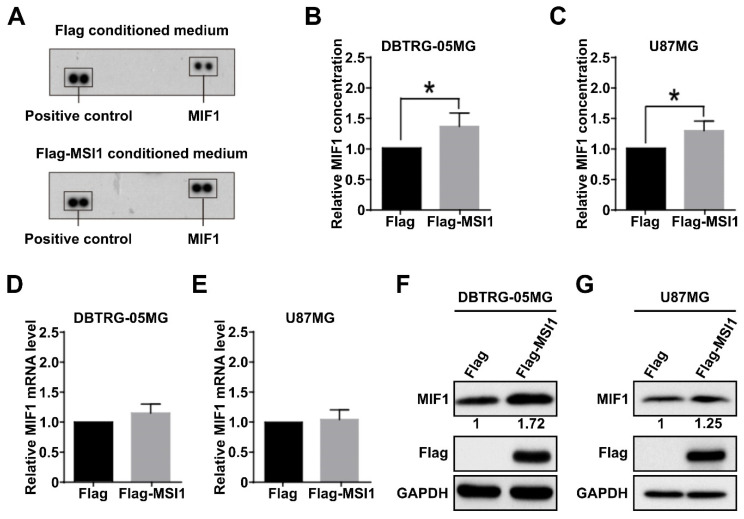
Musashi-1 overexpression in GBM cells elevates MIF1 expression and secretion. (**A**) Cytokine array analysis showing the level of MIF1 in the medium conditioned by the DBTRG-05MG cells overexpressing Flag–MSI1 (bottom panel). The top panel shows results from the cells transfected with a control Flag-only plasmid. (**B**,**C**) ELISA analysis showing the levels of MIF1 in the medium conditioned by the DBTRG-05MG (**B**) or U87MG (**C**) cells overexpressing Flag–MSI1. Mean values relative to the Flag-only control are shown, *n* = 3, SD—error bars, * *p* < 0.05 (Student’s *t*-test). (**D**,**E**) Quantitative RT-PCR analysis showing the expression of *MIF1* mRNA in the DBTRG-05MG (**D**) or U87MG € cells overexpressing Flag–MSI1. Mean values relative to the Flag-only control are shown, *n* = 3, SD—error bars. (**F**,**G**) Western blotting showing the protein level of MIF1 in the DBTRG-05MG (**F**) or U87MG (**G**) cells overexpressing Flag–MSI1. Numbers under the top panel show densitometry quantification of MIF1 bands. GAPDH was used as a loading control.

An increasing number of reports have documented the importance of various subtypes of macrophages in the tumor microenvironment [35,36]. To identify cytokines whose secretion may be important for recruitment of macrophages into the GBM microenvironment in an MSI1-dependent manner, the latter was transiently overexpressed in the DBTRG-05MG cell line. The conditioned medium was exposed to cytokine array analysis, and it was revealed that MSI1 overexpression resulted in upregulation of the cytokine macrophage inhibitory factor 1 (MIF1) (Figure 1A). Furthermore, significant increase of MIF1 secretion was also detected by ELISA in the DBTRG-05MG (Figure 1B) and U87MG cells (Figure 1C) overexpressing MSI1. To further test the mechanism of MSI1-dependent regulation of MIF1 expression, we performed qRT-PCR and Western blotting analysis. Whereas the mRNA levels of *MIF1* did not change upon MSI1 overexpression in either the DBTRG-05MG (Figure 1D) or U87MG cells (Figure 1E), the protein levels were noticeably elevated in both cell lines (Figure 1F,G). Moreover, silencing of MSI1 led to a largely decreased MIF1 level detected by the cytokine array (Supplementary Figure S1). To summarize, we demonstrate that MSI1 regulates expression and, as a result, secretion of MIF1 by regulating its translation.

### 2.2. THP-1-Derived Macrophages Are Polarized into the M2 State in the MSI1-Dependent Manner

**Figure 2:**
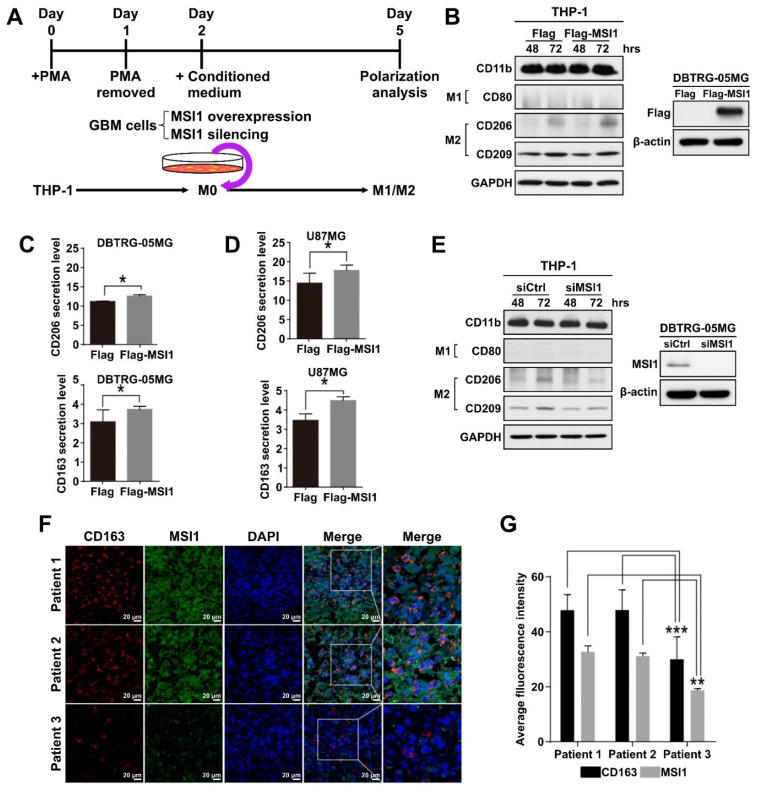
THP-1-derived macrophages are polarized into the M2 state in the MSI1-dependent manner. (**A**) Schematic diagram of the experimental design to test the effect of the GBM cell-conditioned media on macrophage polarization. (**B**) Left panel: immunoblotting analysis of the expression of the indicated M1 and M2 markers in the THP-1-derived macrophages treated with the media conditioned by the DBTRG-05MG cells transfected with Flag–MSI1 and the control Flag-only plasmid (Flag) for 48 and 72 h. Right panel: immunoblotting analysis of the expression of Flag–MSI1 in transfected DBTRG-05MG cells. GAPDH and β-actin were used as loading controls. (**C**,**D**) ELISA analysis of the secretion of CD206 and CD163 by the THP-1-macrophages treated with the media conditioned by the DBTRG-05MG (**C**) and U87MG (**D**) cells transfected with Flag-MSI and the control Flagonly plasmid (Flag). Mean values are shown, *n* = 3, SD—error bars, * *p* < 0.05 (Student’s *t*-test). (**E**) Immunoblotting analysis of the expression of the indicated M1 and M2 markers in the THP-1-derived macrophages treated with the media conditioned by the DBTRG-05MG cells subjected to MSI1 knockdown. The right panel shows immunoblotting analysis of MSI1 expression upon knockdown. (**F**) Immunofluorescent staining of CD163 and MSI1 in cross-sections of three patients’ GBM samples, nuclei stained with DAPI. (**G**) Quantification of the fluorescent signal in (**F**) was achieved by ImageJ measuring of the staining intensity with five views measured for each sample. Mean values are shown, *n* = 3, ** *p* < 0.01, *** *p* < 0.05 (Student’s *t*-test).

In the tumor microenvironment, macrophage polarization leads to two distinct phenotypes, M1 and M2, with the M2 phenotype promoting tumor growth. Therefore, we established a macrophage polarization model using GBM cell lines and THP-1 monocyte cells. In this model, human THP-1 monocytes were first differentiated into M0 macrophages by incubation with phorbol 12-myristate 13-acetate (PMA) until the CD11b marker was upregulated (Figure 2A, Supplementary Figure S2A). The THP-1-derived M0 macrophages were then treated with conditioned media generated from the DBTRG-05MG cells overexpressing exogenous MSI1 or with silenced endogenous MSI1, and the M1/M2 polarization status was then analyzed after allowing differentiation to take place for three days (Figure 2A). The completion of M1 or M2 polarization was validated by examining the expression of CD80 or CD163, CD206, CD209 markers, respectively. Indeed, the control M1 and M2 macrophages induced in parallel to polarize to their respective states by standard protocols exhibited proper expression of their respective markers (Supplementary Figure S2B,C). After incubation with the conditioned medium from the MSI1-overexpressing DBTRG-05MG cells, THP-1 monocytes were preferentially differentiated into M2 macrophages, as the CD80 protein expression was barely detected, while CD206 and CD209 were largely increased when compared to the cells treated with the medium conditioned by the DBTRG-05MG cells transfected with the Flag-only control plasmid (Figure 2B). Similarly, the endogenous expression of CD206 was also increased in macrophages induced by MSI1-overexpressing U87MG-conditioned medium (Supplementary Figure S2D). The ELISA analysis of cytokines released into the culture medium also showed significantly increased CD206 and CD163 secretion by macrophages induced to polarize by the media conditioned by the MSI1-overexpressing DBTRG-05MG (Figure 2C) and U87MG (Figure 2D) cells. In contrast, silencing of endogenous MSI1 by siRNA led to lowered expression of CD206 when compared to that of siRNA control in DBTRG-05MG cells (Figure 2E). Interestingly, the immunofluorescence analysis of the expression of CD163 and MSI1 in three clinical GBM specimens revealed that the higher expression of the former was associated with the higher expression of the latter indicative of possible correlation in vivo (Figure 2F,G). In conclusion, we show that MSI1 expression in GBM cells results in secretion of the factor/factors that cause polarization of macrophages to the M2 type.

### 2.3. MSI1 Expression Is Highly Correlated with MIF1 in GBM Tumor Mouse Models

**Figure 3:**
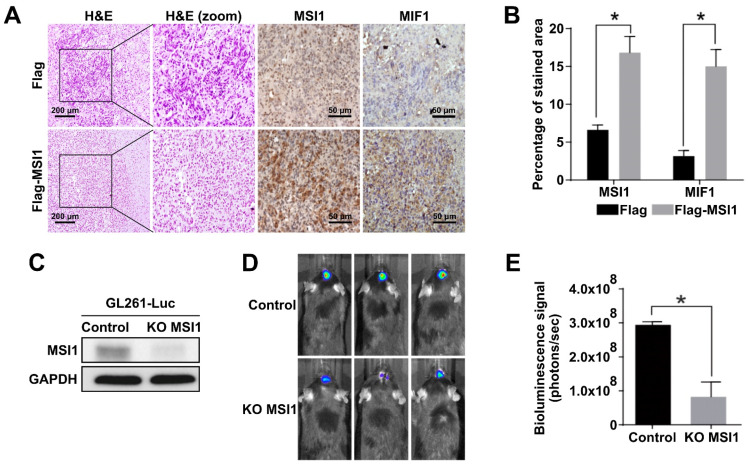
MSI1 expression is highly correlated with MIF1 in GBM tumor mouse models. (**A**) Immunohistostaining of MSI1 and MIF1 in cross-sections of intracranial tumors derived from xenografts of the DBTRG-05MG cells transfected with Flag–MSI1 and the control Flag-only construct. H&E staining (left panels) shows general tumor morphology. (**B**) Quantification of MSI1—and MIF1—stained areas in (**A**). Mean values are shown, *n* = 3, SD—error bars, * *p* < 0.05 (Student’s *t*-test). (**C**) Western blot showing the knockout of MSI1 expression in the GL261-Luc cells transfected with Cas9 only (control) or with CRISPR/Cas9 (KO MSI1). GAPDH—loading control. (**D**) Control and KO MSI1 GL261-Luc cells were subjected to an intracranial injection in 8-week-old male C57BL/6 mice (*n* = 3). Bioluminescent signal visualization (IVIS) of tumors derived from control and KO MSI1 GL261-Luc cells in mice on day 14 post-injection is show€(**E**) Quantification of the bioluminescence signal from tumors in (**D**); *n* = 3, SD—error bars, * *p* < 0.05 (Student’s *t*-test).

To further investigate the interplay between MSI1 and MIF1 in vivo, we established a mouse GBM xenograft model [37]. For this purpose, the MSI1-overexpressing DBTRG-05MG cells were intracranially xenografted into immunocompromised mice and the expression of MIF1 and MSI1 was interrogated by immunostaining. Our data demonstrated that the level of MIF1 was indeed significantly elevated in the tumor tissues derived from the MSI1-overexpressing DBTRG-05MG cells (Figure 3A,B). The in vivo consequences of MSI1-mediated MIF1 enrichment were further substantiated by utilizing GL261-Luc, another frequently used syngeneic murine GBM model that expresses luciferase to facilitate visualization of tumor growth and responses to treatments via bioluminescence imaging [38]. At first, we used the CRISPR/Cas9 technology to knock out the *MSI1* gene in the GL261-Luc cell line (Figure 3C). As a result, it was shown that *MSI1* knockout led to significantly smaller intracranial tumors in this syngeneic mouse model (Figure 3D,E). To summarize, we confirm that MSI1 overexpression leads to MIF1 upregulation in vivo and that such interplay between these two factors may result in enhanced tumor growth in an in vivo mouse model.

### 2.4. MIF1 Antagonist ISO-1 Is Effective in Reducing the M2 Subpopulation and Cellular Proliferation, Migration and Phagocytosis

**Figure 4:**
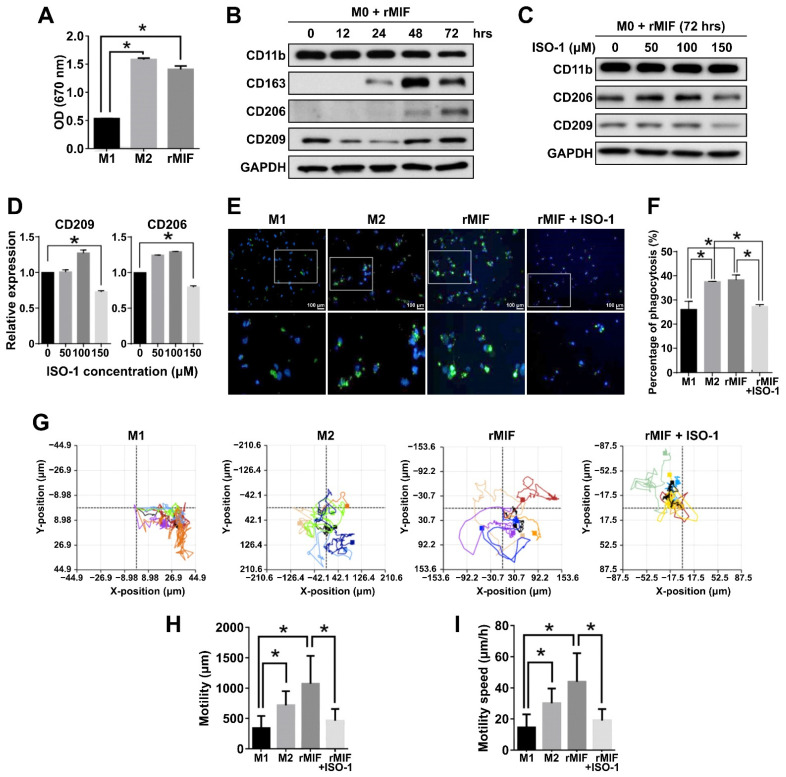
MIF1 antagonist ISO-1 is effective in reducing the M2 subpopulation and cellular proliferation, migration and phagocytosis. (**A**) MTT proliferation assay showing the effect of rMIF on proliferation of the rMIF-induced M0 phagocytes as compared to the M1 and M2 phagocytes derived by standard protocols. Mean values are shown, *n* = 3, SD—error bars, * *p* < 0.05 (Student’s *t*-test). (**B**) Western blotting showing the expression of the indicated macrophage markers in the time course of treatment of M0 macrophages with 100 ng/mL rMIF. GAPDH—loading control. (**C**) Western blotting analysis of the expression of the indicated markers in the cells derived by treatment of M0 macrophages with rMIF for 72 h in the absence (0 μM) or presence of ISO-1 at the indicated concentrations. (**D**) Densitometry quantification of the expression of CD209 and CD206 shown by Western blotting in (**C**). Mean values relative to 0 μM ISO-1 are shown, *n* = 3, SD—error bars, * *p* < 0.05. (**E**) Fluorescence microscopy images showing phagocytotic cells (green fluorescence) among the indicated macrophages. The bottom panel shows a zoomed view of the areas marked by rectangles in the top panel. Nuclei stained with DAPI (blue fluorescence). (**F**) Quantitative analysis showing the percentage of phagocytotic macrophages among the total number of macrophages. Means with SD error bars are shown, *n* = 3, * *p* < 0.05 (Student’s *t*-test). (**G**) Single-cell tracking by time-lapse microscopy of the indicated groups of macrophages. (**H**,**I**) Quantification of motility (**H**) and motility speed (**I**) of the indicated macrophages measured by time-lapse microscopy in (**G**). Mean values with SD error bars are shown, *n* = 3, * *p* < 0.05 (Student’s *t*-test).

To further corroborate biological functions of MSI1-mediated MIF1 expression in GBM, we examined whether MIF1 antagonist ISO-1 could exert any impact on polarization of THP-1 cells. First, THP-1 cells were differentiated or treated with recombinant MIF1 protein (rMIF), and proliferation of M1 and M2 THP-1-derived macrophages obtained by standard protocols as well as of the rMIF-treated THP-1 cells was measured. The result showed that the rMIF-treated cells were able to proliferate as fast as M2 macrophages and significantly faster than M1 macrophages (Figure 4A). Time course of rMIF treatment showed consistently increased expression of CD206 after 72 h, as well as CD163 upregulation after 24 h in the rMIF-treated macrophages (Figure 4B). MIF antagonist ISO-1 was next used at different concentrations to test whether it could avert rMIF-mediated macrophage differentiation. Indeed, the expression of both CD206 and CD209 markers induced by 72 h of rMIF treatment was markedly decreased in the presence of 150 μM ISO-1 (Figure 4C). Macrophage polarization causes functional changes in their behavior. It has been reported that the ability of phagocytosis and migration in M2 macrophages is higher than in M1-type macrophages [39,40]. Therefore, we tested these functional properties in our experimental system. Indeed, treatment of M0 macrophages with rMIF led to significant increase of phagocytosis, which was comparable to that in M2 macrophages; however, this effect was reversed by concomitant treatment with ISO-1 (Figure 4E,F). Besides, to measure the migration capacity of the rMIF-induced macrophages, a single-cell migration trace recording was collected for 24 h (Figure 4G). We showed that the motility and motility speed of the MIF-induced macrophages were increased and were similar to the trend observed in M2 macrophages; however, the concomitant treatment with ISO-1 resulted in a reduced motility more similar to that of M1 macrophages (Figure 4G–I). To summarize, these functional assays revealed that the MIF-induced macrophages belonged to the M2-like phenotype and promoted phagocytotic activity and migration ability.

### 2.5. Inhibition of MIF1 Causes Suppression of GBM Growth In Vivo with Concomitant Reduction of the M2 Macrophage Population

**Figure 5:**
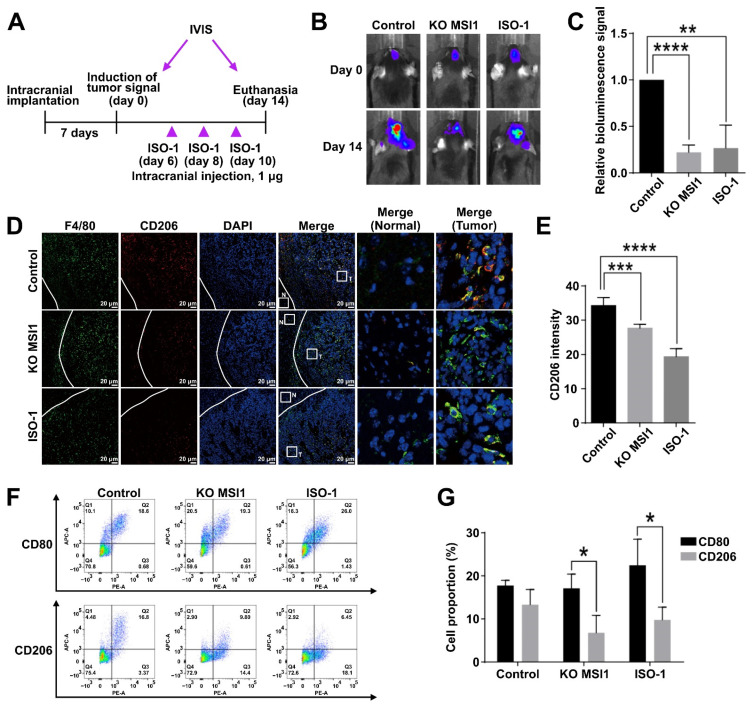
Inhibition of MIF1 causes suppression of GBM growth in vivo with concomitant reduction of the M2 macrophage population. (**A**) Schematic of the experimental design to test the effect of ISO-1 on intracranial tumor growth. GL261-Luc cells were intracranially implanted in mice (*n* = 3) and inoculated for seven days until the signal of tumor induction was observed by the IVIS luciferase imaging system (defined as day 0). The MIF-1 inhibitor ISO-1 (1 μg) was intracranially injected at days 6, 8 and 10. The signals of intracranial tumors were monitored by the IVIS luciferase imaging system till day 14 when the mice were sacrificed. (**B**) Bioluminescent imaging visualization (IVIS) of the indicated GL261-Luc-derived tumors. (**C**) Quantification of the bioluminescence signal from tumors in (**B**). Means with SD error bars are shown, *n* = 3, ** *p* < 0.01, **** *p* < 0.0001 (Student’s *t*-test). (**D**) Immunofluorescent staining of F4/80 (general macrophage marker) and CD206 (M2 marker) in the cross-sections of the indicated GL261-Luc-derived intracranial tumors. Tumor (T) and surrounding normal (N) tissue are marked by white squares and zoomed in the right panel. (**E**) Quantification of the CD206 immunofluorescent signal intensity in (**D**). Means are shown with SD error bars, *n* = 3, *** *p* < 0.005, **** *p* < 0.0001 (Student’s *t*-test). (**F**) Flow cytometry analysis of the proportion of CD80 (M1)- and CD206 (M2)-expressing macrophages in the indicated GL261-Luc-derived intracranial tumors. (**G**) Quantification of the proportion of CD80- and CD206-expressing macrophages in (**F**). Mean values are shown with SD error bars, *n* = 3, * *p* < 0.05 (Student’s *t*-test).

After confirming the antioncogenic effects of MIF1 inhibition in vitro, we applied a GL261-Luc syngeneic mouse model to test the effects of the ISO-1 inhibitor in vivo. For this purpose, immunocompromised mice were implanted with wildtype GL261-Luc cells in parallel with the cells subjected to the knockout of the *MSI1* gene. After the initial tumor growth, the initial IVIS bioluminescence recordings of the GL261-derived intracranial tumors were made, and within the next two weeks, three administrations of ISO-1 were performed to a batch of mice intracranially transplanted with the wildtype GL261-Luc cells (Figure 5A). As was observed by bioluminescence recording after two weeks, the ISO-1-treated mice demonstrated noticeably stunted tumor growth compared to the untreated control mice (Figure 5B,C). The scale of this intracranial tumor growth reduction was the same as demonstrated by tumors derived from *MSI1* knockout GL261-Luc cells (Figure 5B,C). Remarkably, as was shown by immunofluorescence staining, CD206 marker was severely reduced by both *MSI1* knockout and ISO-1 administration, indicating reduction of the M2 macrophage population (Figure 5D,E). Further analysis by flow cytometry showed that while the CD80-expressing M1 macrophage population was not affected by either ISO-1 treatment or *MSI1* knockdown, the CD206-expressing M2 population was markedly reduced (Figure 5F,G). To conclude, we show that inhibition of MIF1 by ISO-1 reduces GBM growth in vivo with concomitant reduction of the M2 macrophage population in a manner similar to that caused by the knockout of *MSI1*.

## 3. Discussion

MSI1 is an RNA-binding protein (RBP) which is abundantly found in neural stem cells, as well as highly expressed in GBM [41]. Besides, GBM microenvironment is usually highly infiltrated by tumor-associated macrophages (TAMs) which may promote GBM growth [42]. According to our findings, we determined the correlation between MSI1 and TAMs in GBM. We also discovered that the macrophage inhibitory factor 1 (MIF1) expression is positively regulated by MSI1 in vitro and in vivo. Overexpression of exogenous MSI1 increased the MIF1 protein level in GBM cells, while the mRNA level was not significantly changed indicating positive translational regulation of MIF1 by MSI1 (Figure 1). Originally, MSI1 was identified as a negative regulator of translation, e.g., its binding to the 3′-UTR of the *NUMB1* mRNA and interaction with poly(A)-binding protein (PABP) was shown to interfere with cap-mediated initiation of translation [10]. On the other hand, in different contexts, MSI1 interaction with PABP was also shown to positively regulate translation [43]. Therefore, the latter mechanism is highly plausible to be implicated in the regulation of MIF1 by MSI1.

Recent evidence indicates that M2 macrophages contribute to GBM progression and growth [22]. In our study, we showed that M2 macrophages polarized from M0 macrophages were capable of further elevating cellular growth of GBM when treated with MIF1. In addition to impacting the GBM proliferation, our transwell migration data also revealed that MIF1 was able to induce cellular metastasis and invasion of GBM cells when cocultured with M1, M2 or MIF1-induced macrophages. Indeed, these observations clearly fit the model of double function of macrophages, whereby M1 macrophages are responsible for wound healing in the normal context, but those normal functions being responsible for tumor growth and malignancy, and M2 macrophages are responsible for immune responses [44].

MIF1 is a well-known inflammatory cytokine that functions by regulating catalytic activity, lymphocyte immunity, endocrine regulation, signal modulation and proinflammatory action. It is also highly expressed in cancers, such as colon cancer, melanoma and GBM [45,46,47]. MIF1 was also found to contribute to tumor angiogenesis and promotion of cell cycle progression through abolishing tumor suppressive activity of p53 [48]. In line with those observations, our data demonstrated that MIF1-mediated M2 macrophage polarization did not only exert suppression effects on GBM cells in vitro, but also led to significant tumor suppression in our GBM tumor models. The impact of MIF1 in GBM was further reinstated by administration of the MIF1-specific inhibitor, ISO-1, in our orthotopically implanted GL-261-Luc GBM model. The ISO-1-treated GMB model demonstrated not only significantly reduced tumor volumes, but also consistently decreased CD206 expression and M2 macrophage population.

Recently, increasing research efforts have been made into identifying small molecules that target the tautomerase active site of MIF as well as anti-MIF antibodies for neutralization of MIF [49,50]. Our data on ISO-1 as a profound GBM tumor suppressor via MSI1-mediated MIF inhibition have thus lent further support for the development of ISO-1 as a therapeutic for treating GBM. Our findings of the higher expression of MSI1/MIF in the GBM tissue when compared with the normal tissue suggested that MSI1 and MIF may act as diagnostic markers for clinical GBM malignancies.

Moreover, despite the mechanism underlying MIF-induced M2 macrophage polarization by MSI1 remains unclear, MSI1 was shown to correlate with drug resistance in GBM under AKT- and IL-6-mediated malignancies in one of our previous studies [33]. Whether there was any interplay between MSI1/MIF/M2 macrophages and the MSI1/AKT/IL-6 axis requires further investigation. In fact, a study by Lue et al. showed that MIF could bind with MIF receptor CD74 to activate MERK/ERK or PI3K/Akt-dependent signaling pathways in cervical and breast cancer cells [51]. Thus, molecular approaches undertaken in the current study including MSI1-specific knockdown as well as MSI1/MIF-mediated M2 polarization have paved the way to delineating the signaling mechanism that underlines the phenomenal GBM tumor suppression effects observed.

In summary, our current study utilized the cytokine array and identified the pivotal role of MSI1 in the MIF-mediated M2 macrophage differentiation in the tumor progression of GBM. The MSI1/MIF1/M2 macrophage axis may thus serve as a new opportunity of drug discovery for the treatment or diagnostics of GBM in clinics.

## 4. Materials and Methods

### 4.1. Cell Culture and Clinical Tissue

Human GBM cell lines DBTRG-05MG and U87MG and a mouse GL261-Luc GBM cell line were acquired from the American Type Culture Collection (ATCC). DBTRG-05MG, U87MG and GL261-Luc cells were maintained in a DMEM (high-glucose) medium supplemented with 10% fetal bovine serum and 1% penicillin/streptomycin in a 5% CO_2_ incubator at 37 °C. THP-1 cells were maintained in an RPMI medium (Sigma-Aldrich, St Louis, MO, USA) supplemented with 10% fetal bovine serum and 1% penicillin/streptomycin in a 5% CO_2_ incubator at 37 °C. MIF inhibitor, (S,R)-3-(4-hydroxyphenyl)-4,5-dihydro-5-isoxazole acetic acid (ISO-1), was purchased from Merck Millipore (Burlington, MA, USA). All the cell lines were tested for mycoplasma contamination. The clinical tissue samples were acquired from the Neurological Institute of Taipei Veterans General Hospital. All procedures of tissue acquisition followed the tenets of the Declaration of Helsinki and were reviewed by the Institutional Review Committee at Taipei Veterans General Hospital (ethics authorization number: 2016-09-012C).

### 4.2. Differentiation and Polarization of THP-1 Cells

For the differentiation and polarization of THP-1 monocytes into M1 and M2 macrophages, they were first incubated for 24 h with 20 nM phorbol-12-myristate-13-acetate (PMA) in an RPMI medium supplemented with 10% fetal bovine serum and 1% penicillin/streptomycin in a 5% CO_2_ incubator at 37 °C. After that, the cells were rinsed with PBS once and a fresh RPMI culture medium without PMA was added for the next 24 h. Macrophages were polarized into the M1 type by incubation with 20 ng/mL IFN-γ and 10 ng/mLf LPS. M2 macrophage polarization was induced by incubation with 20 ng/mL interleukin 4 (IL-4) and 20 ng/mL interleukin 13 (IL-13). Recombinant MIF protein was purchased from PeproTech (Rocky Hill, NJ, USA).

### 4.3. Animals and GBM Tumor Models

All the experiments were performed in accordance with the guidelines set forth by the European Community’s Council Directive of 24 November 1986 (86/609/EEC). The study was approved according to the institutional animal welfare guidelines of Taipei Veterans General Hospital, Taiwan, on the control of the maintenance and use of animals (protocol number: 2019-011; 1 August 2019). For intracranial transplantation, DBTRG-05MG human glioma and GL261-Luc murine glioma cell lines and their derivatives were harvested, washed with PBS and spun down to remove excess PBS. A total volume of 2.5 μL containing 5 × 10^5^ cells was injected orthotopically into the brain of 8-week-old male C57BL/6 mice (National Laboratory Animal Center, Taipei, Taiwan) bred and maintained according to the Guidelines for Laboratory Animals at Taipei Veterans General Hospital. Intracranial tumor growth was monitored up to 14 days after injection by bioluminescent imaging with a Xenogen IVIS 50 imaging system following intraperitoneal injection with 250 μL of 15 mg/mL stock solution of D-Luciferin K salt (Gold Biotechnology, Olivette, MO, USA). The animals were humanitarianly sacrificed 14 days after tumor induction.

### 4.4. Plasmid Construction and Transfection

MSI1 coding sequence was amplified and subcloned from human cDNA using the following primers with the introduced restriction sites: MSI1-F-HindIII (AGAAGCTTATGGAGACTGACGCGCCCCAGC) and MSI1-R-BamHI (AGGATCCTCAGTGGTACCCATTGGTGAAGG). Plasmids p3XFlag–MSI1 and pmOrange-MSI1 were generated by inserting a 1038-bp amplified fragment of the full-length human MSI1 coding sequence into the HindIII/BamHI site of the p3XFlag-myc-CMV-26 and pmOrange vectors, respectively. Gene delivery by plasmid transfection was carried out using a jetPEI DNA transfection reagent (Polyplus-transfection, New York, NY, USA) according to the manufacturer’s instructions. The siRNA against MSI1 (SASI_Hs01_00145278) and scrambled siRNA control (MISSION siRNA Universal Negative Control #1) were purchased from Sigma-Aldrich. Transient siRNA transfection was carried out using INTERFERin siRNA transfection reagent (Polyplus-transfection) according to the manufacturer’s instructions.

### 4.5. Polymerase Chain Reaction

Total RNA was extracted using a TRIzol reagent (Thermo Fisher Scientific, Waltham, MA, USA) according to the manufacturer’s instructions. Total RNA (1 μg) was converted to cDNA with random primers and a SuperScript III reverse transcriptase (ThermoFisher Scientific) according to the manufacturer’s protocol. For quantitative real-time polymerase chain reaction (qRT-PCR), cDNA was diluted to 200 ng/μL with sterile water and mixed with a SYBR Green PCR Master Mix (ThermoFisher Scientific) and primers. Amplification reactions were performed using a 7900HT Fast Real-Time PCR System (ThermoFisher Scientific) according to the manufacturer’s instructions. Messenger RNA abundance was quantified according to the ΔΔC_T_ method and 18S was used as an internal control for normalization. The following primer pairs were used: 18S-F: CAGCCACCCGAGATTGAGCA; 18S-R: TAGTAGCGACGGGCGGTGTG; MSI1-F: ACCGAGGGTTCGGGTTTGTC; MSI1-R: GCCGATGCCCAGCATGAAGG; CD80-F: GCAGGGAACATCACCATCCA; CD80-R: TCACGTGGATAACACCTGAACA; CD206-F: GGGAAAGGTTACCCTGGTGG; CD206-R: TCAAGGAAGGGTCGGATCGT; CD209-F: CTAAAGCAGGAGTTCTGGAC; CD209-R: CTAAAGGTCGAAGGATGGAG; MIF1-F: CGGACAGGGTCTACATCAACT; MIF1-R: TTCTCCCCACCAGAAGGTTG.

### 4.6. Protein Extraction and Western Blotting

The culture medium was removed and the cells were washed with PBS; total cellular proteins were extracted using a RIPA buffer with protease inhibitors. After 10-min incubation on ice, the cell lysates were centrifuged at 13,000 rpm for 15 min at 4 °C, and supernatants containing proteins were collected and stored at −80 °C before being analyzed by Western blotting using SDS polyacrylamide gel electrophoresis. Soluble protein fraction and protein marker (30 μg) were loaded per lane on 8–13% polyacrylamide gels. After being stacked at 75 V and separated at 120 V, the protein samples were then transferred onto polyvinylidene fluoride (PVDF) membranes at 90 V for 3 h. The PVDF membranes were blocked in 5% skim milk in PBST for 1 h followed by hybridizing with primary antibodies overnight at 4 °C. After incubation with primary antibodies, the PVDF membranes were washed three times in PBST for 10 min before incubation with an HRP-conjugated rabbit/mouse secondary IgG for 1 h at room temperature. The membrane was washed three times for 10 min and signals were visualized with an enhanced chemiluminescence (ECL) kit.

### 4.7. Immunofluorescent Staining

Patient specimens (*n* = 3) were deparaffinized, rehydrated and subjected to antigen retrieval by boiling in a retrieval buffer for 15 min. The sections were cooled in PBS for 10 min. The samples were blocked in 5 mg/mL BSA for 1 h before hybridizing with 1:200 diluted primary antibodies against MSI1 and CD163 at 4 °C overnight, and then Alexa Fluor 488- or 555-conjugated secondary antibodies were added at 1:200 dilution and the samples were incubated at room temperature for 1 h. The sections were then washed twice in PBS and stained with DAPI. Sample slices were flat-mounted with a mounting solution. Immunohistochemistry (IHC) tumor specimens from mice were fixed with 4% paraformaldehyde. The sections were deparaffinized, rehydrated and subjected to antigen retrieval by boiling in a retrieval buffer for 15 min. The sections were cooled in PBS for 10 min. The samples were blocked in 5 mg/mL BSA for 1 h before hybridizing with 1:150 diluted primary antibodies (MIF1, MSI1 and CD206) at 4 °C overnight. The signals were amplified by a Polymer-HRP IHC Detection system (BioGenex, San Ramon, CA, USA) following the manufacturer’s instructions. The sections were examined under an Olympus BX61 microscope.

### 4.8. Flow Cytometry

Mouse tumors were digested in 0.25% trypsin/EDTA without phenol red at 37 °C for 10 min. Digestion was terminated by adding two volumes of an RPMI medium containing 10% FBS. The cells were passed through a 75-μm cell strainer and centrifuged at 800 *g* for 10 min at 4 °C. Cell pellets were resuspended in 30% Percoll (GE Healthcare, Chicago, IL, USA) diluted in an RPMI medium with 1% FBS and layered above 70% Percoll diluted in PBS. The cells were separated by centrifuging at 800 *g* for 30 min at 4 °C. The cells from the 30%/70% Percoll interphase were collected and washed with a FACS buffer (Dulbecco’s phosphate-buffered saline (DPBS) with 0.5% BSA). After centrifugation, the cells were blocked with a blocking buffer (2% FBS in DPBS) on ice for 30 min and stained with F4/80-PE, CD80-APC or CD206-APC antibodies (BioLegend, San Diego, CA, USA). All the data were collected on a BD LSRFortessa flow cytometer (BD, Franklin Lakes, NJ, USA) and analyzed using the FlowJo v10 software.

### 4.9. Cytokine Assays

Cytokine secretion into the culture medium was assayed using a Proteome Profiler Human Cytokine Array kit (R&D Systems, Minneapolis, MN, USA) according to the experimental procedures recommended by the supplier. MIF secretion was measured using a LEGEND MAX Human Active MIF ELISA Kit according to the manufacturer’s protocol (BioLegend).

### 4.10. Proliferation Assay

THP-1 cells (8 × 10^4^) were seeded on 24-well transwells (0.4 μm pore size) and polarized into M1/M2 or MIF1-induced macrophages. The lower chamber was seeded with 2 × 10^4^ DBTRG-05MG cells, which were cocultured with M1/M2/MIF-induced macrophages for 72 h. The culture medium was removed before the addition of MTT to a final concentration of 0.5 mg/mL. After incubating for 45 min at 37 °C, the culture medium with MTT was removed and DMSO was added to dissolve purple crystals formed prior to measurements by an ELISA reader at a wavelength of 560 nm (reference wavelength of 670 nm was used).

### 4.11. Phagocytosis Assay

THP-1 cells (2 × 10^4^) were cultured in 24-well plates and polarized into M1/M2 by standard protocols or incubated with rMIF or a conditioned medium. After 48 h, a Latex Beads-Rabbit IgG–FITC complex (Cayman Chemical, Ann Arbor, MI, USA) was added for 24 h. To prepare a Latex Beads-Rabbit IgG–FITC solution, beads were diluted 1:1000 according to the manufacturer’s protocol.

### 4.12. Statistical Analysis

Data are expressed as the means ± SD from at least three independent experiments. The statistical analysis was performed using the Student’s *t*-test. Differences were considered significant at *p* ≤ 0.05.

## 5. Conclusions

In summary, our current study utilized cytokine array and identified the pivotal role of MSI1 in MIF-mediated M2 macrophage differentiation in the tumor progression of GBM. MSI1/MIF1/M2 macrophage axis may thus serve as a new opportunity of drug discovery to treatment or diagnostics for GBM in clinics.

## Data Availability

Not applicable.

## Supplementary Materials

The following are available online at https://www.mdpi.com/article/10.3390/cancers13081799/s1, Figure S1: Cytokine array of control and MSI1-knockdown conditioned medium, Figure S2: Expresion of respective M1 and M2 macrophage markers, Table S1: Antibodies, Table S2: Primer sequences, Table S3: Chemicals.
