# Supplementary material for: Musashi-1 Regulates MIF1-Mediated M2 Macrophage Polarization in Promoting Glioblastoma Progression
Source: Cancers (Basel). 2021 Apr 9;13(8):1799. doi: 10.3390/cancers13081799 (PMC8069545; doi:10.3390/cancers13081799)
Supplement: Supplementary file 1 [file cancers-13-01799-s001.zip › cancers-1125400-non-published.pdf]

Fig. 1F

MIF

Flag

GAPDH

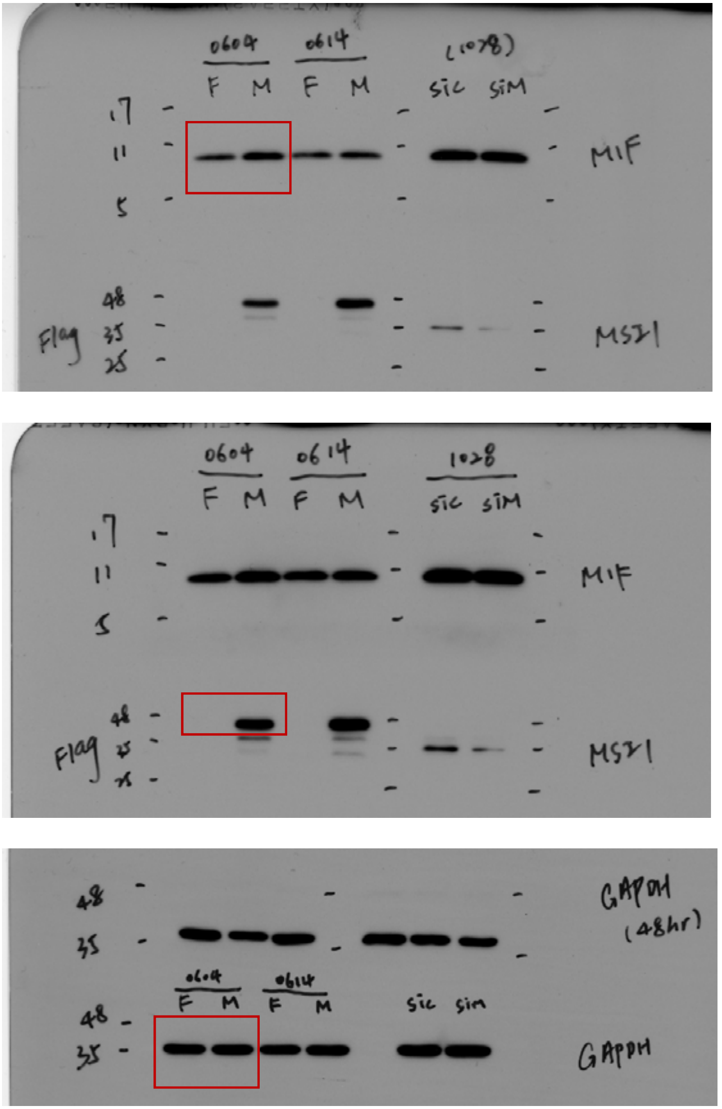

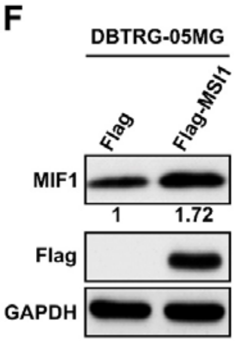

DBTRG-05MG		
	Flag	Flag-MSI1
MIF1	1	1.72
Flag	1	21.75

Fig. 1G

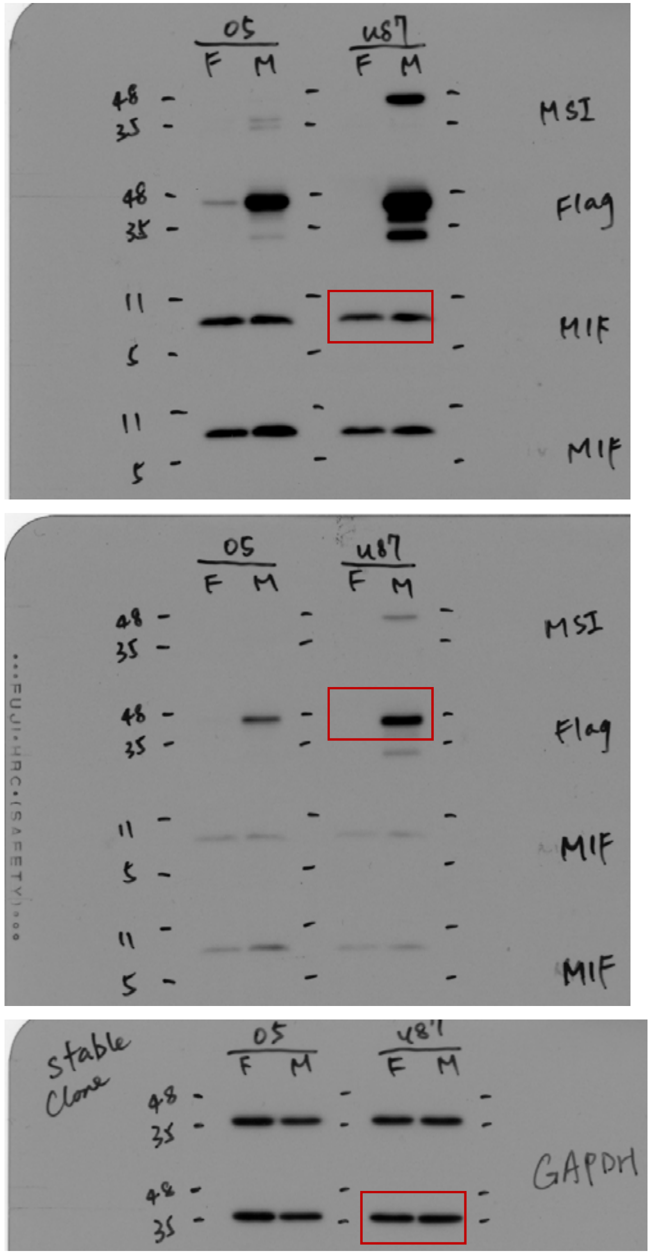

MIF

Flag

GAPDH

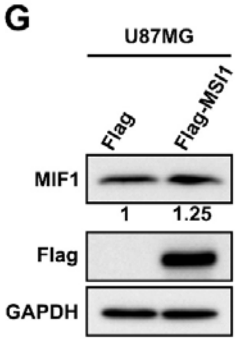

U87MG		
	Flag	Flag-MSI1
MIF1	1	1.25
Flag	1	320.78

Fig. 2B

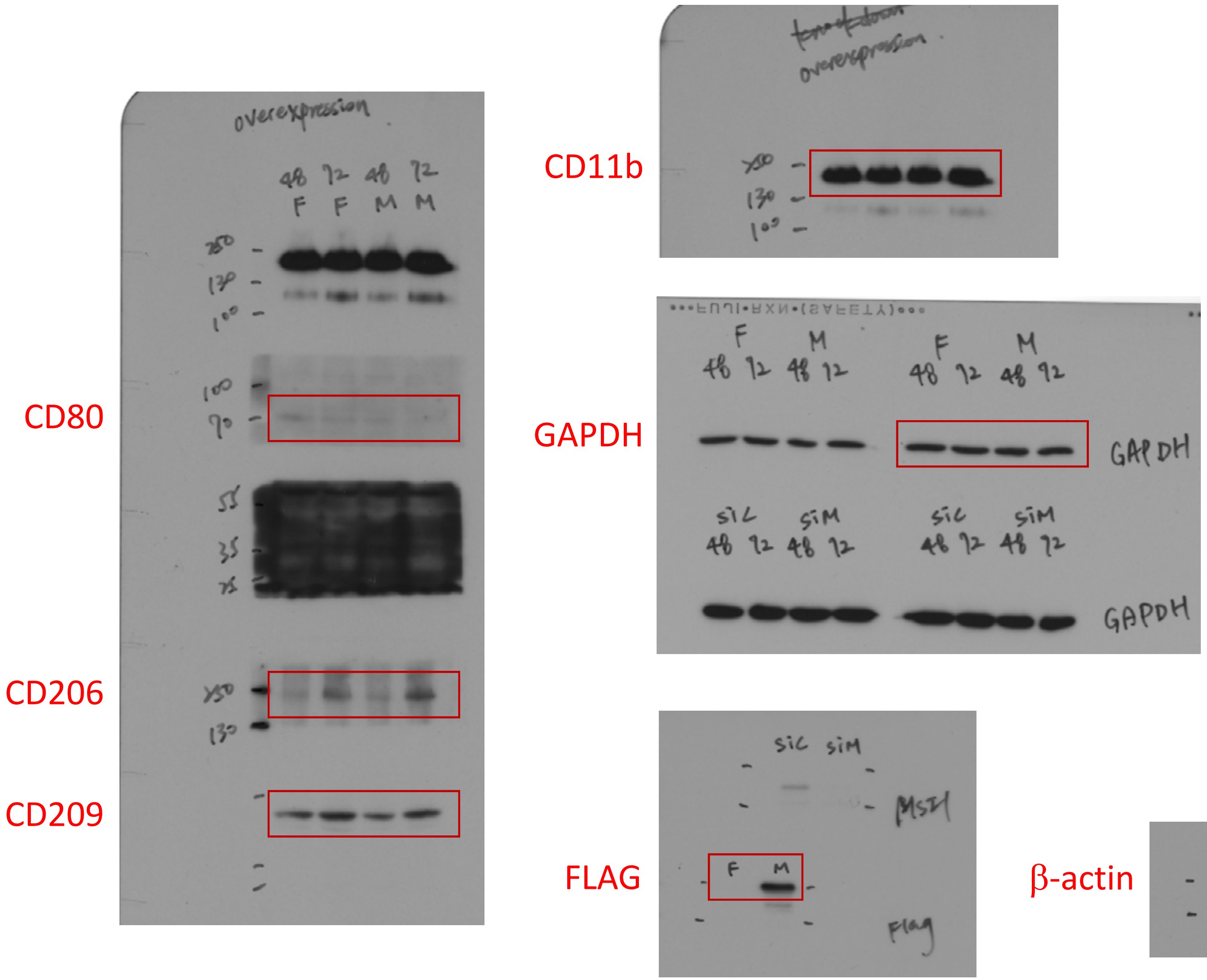

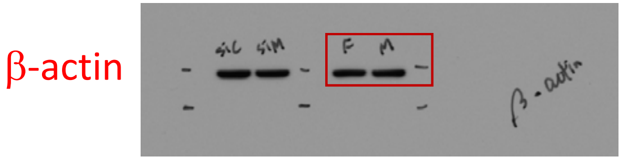

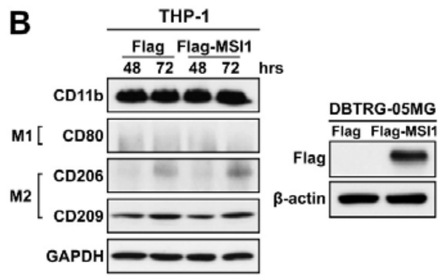

		THP-1			
		Flag		Flag-MSI1	
		48	72	48	72
	CD11b	1	1.09	1.15	1.32
M1	CD80	1	1.06	0.98	0.22
	CD206	1	3.27	1.43	5.42
M2	CD209	1	1.56	0.97	1.52

	DBTRG-05MG	
	Flag	Flag-MSI1
Flag	1	2279.8724

Fig. 2E

CD11b

CD206

CD209

CD80

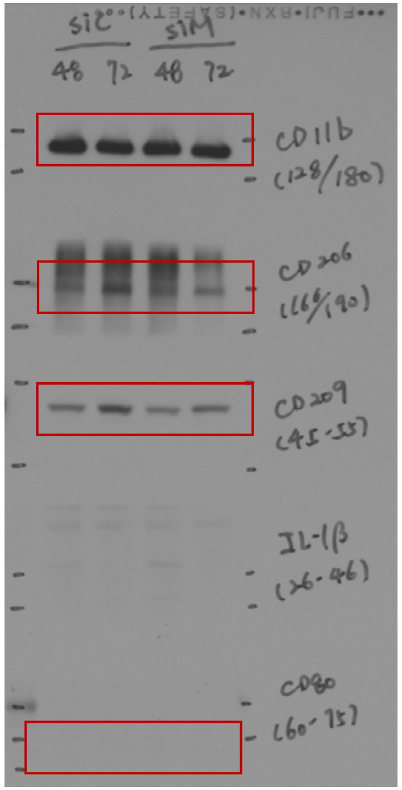

GAPDH

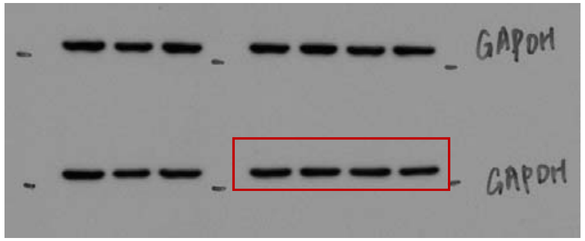

MSI1

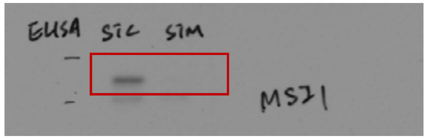

β-actin

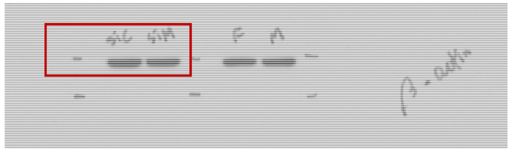

E

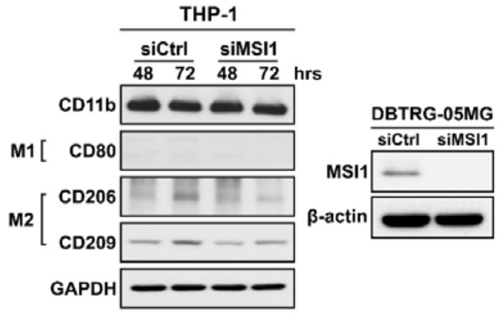

		THP-1			
		siCtrl		siMSI1	
		48	72	48	72
	CD11b	1	0.90	0.99	1.02
M1	CD80	1	1.13	0.86	0.72
	CD206	1	1.68	1.16	0.64
M2	CD209	1	1.86	0.77	1.18

	DBTRG-05MG	
	siCtrl	siMSI1
Flag	1	0.003

Fig. 3C

MSI1

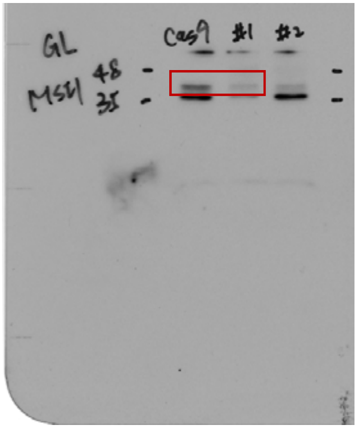

C

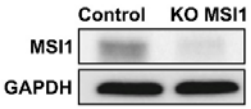

GAPDH

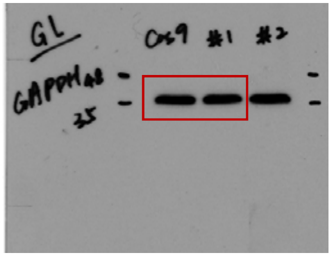

	Control	KO MSI1
MSI1	1	0.18

Fig. 4B

CD11b

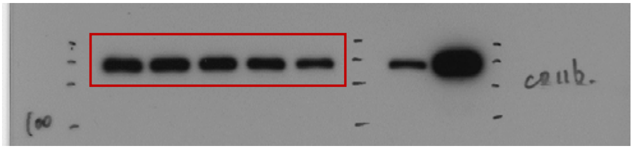

CD163

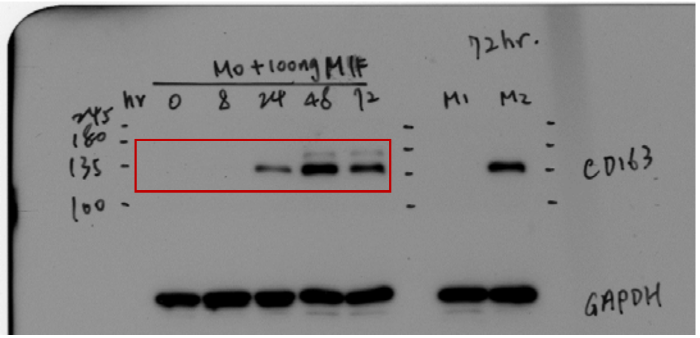

CD206

CD209

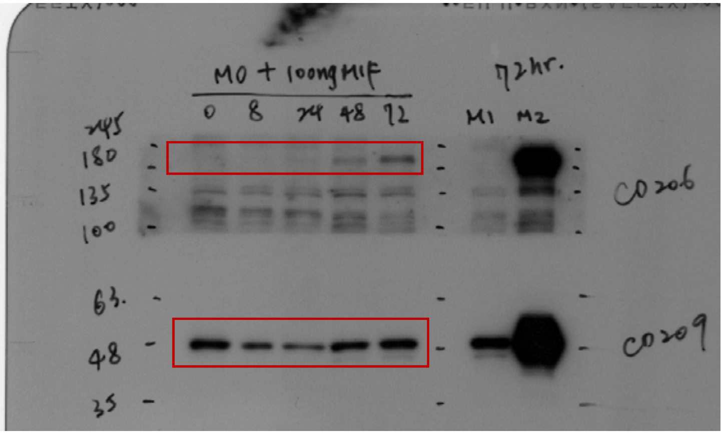

GAPDH

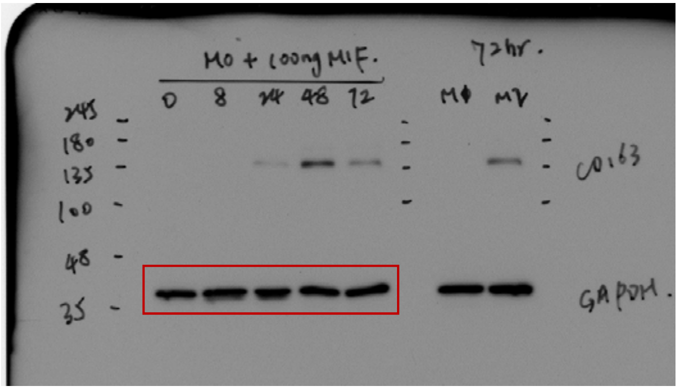

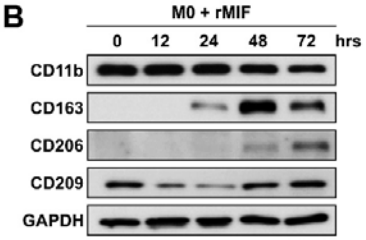

	M0+rMIF				
	0	12	24	48	72
CD11b	1	0.82	0.86	0.74	0.69
CD163	1	2.55	339.76	1049.58	918.96
CD206	1	0.14	0.94	2.58	6.06
CD209	1	0.38	0.38	0.83	1.14

Fig. 4C

CD11b

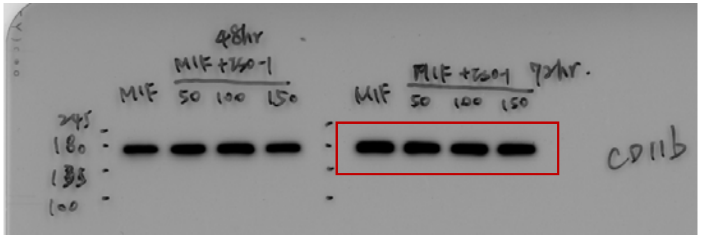

CD206

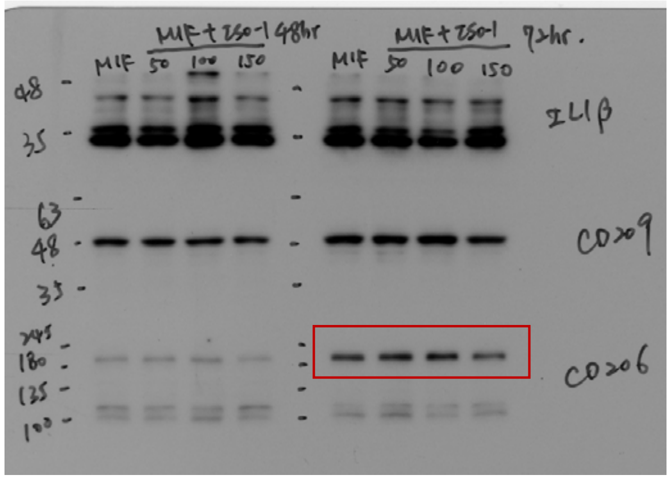

CD209

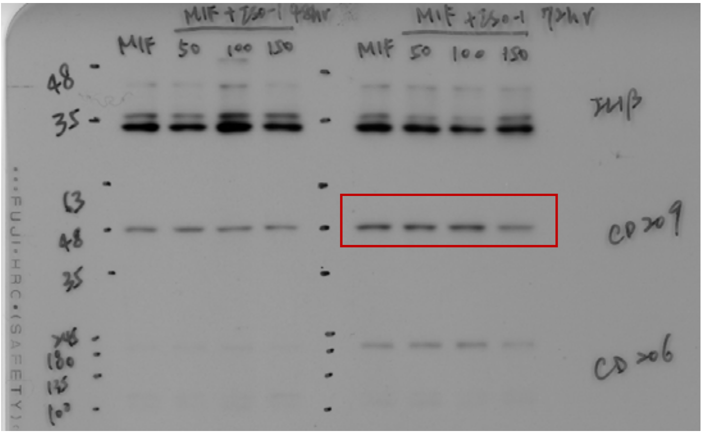

GAPDH

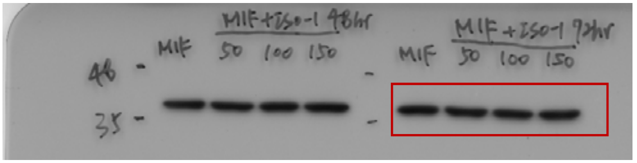

C

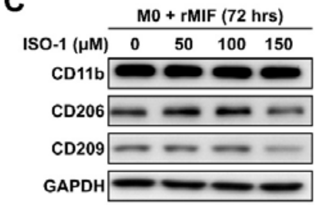

	M0+rMIF (72 hrs)			
ISO-1 (μM)	0	50	100	150
CD11b	1	1.22	1.30	1.11
CD206	1	1.34	1.38	0.77
CD209	1	1.16	1.28	0.66
