# Supplementary material for: Musashi-1 Regulates MIF1-Mediated M2 Macrophage Polarization in Promoting Glioblastoma Progression
Source: Cancers (Basel). 2021 Apr 9;13(8):1799. doi: 10.3390/cancers13081799 (PMC8069545; doi:10.3390/cancers13081799)
Supplement: Supplementary file 1 [file cancers-13-01799-s001.zip › WB raw data-Suppl-2.pdf]

Suppl. Fig. 2A

CD11b

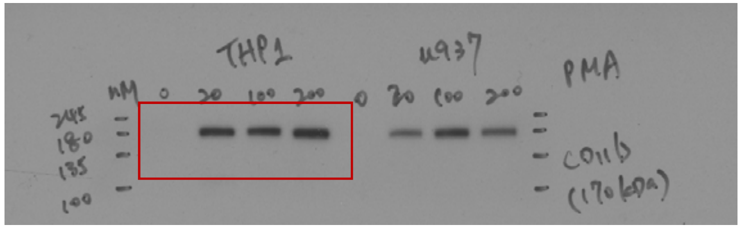

β-actin

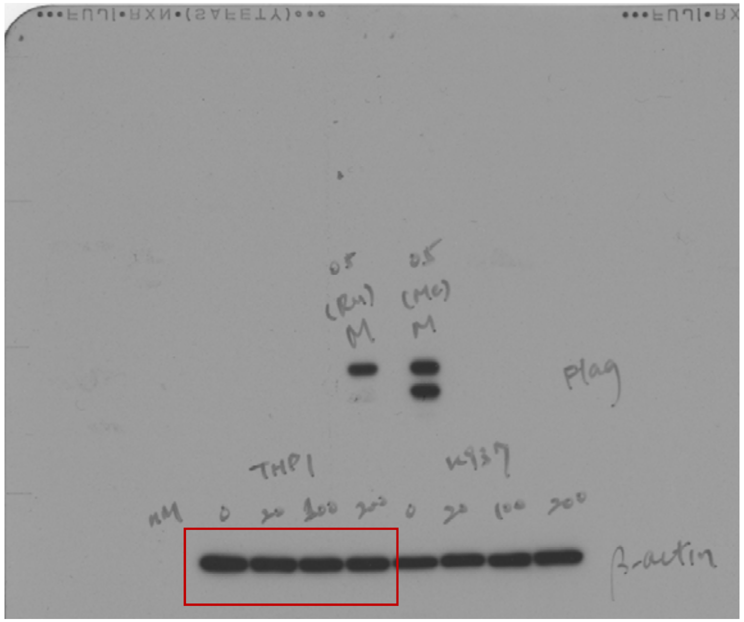

A

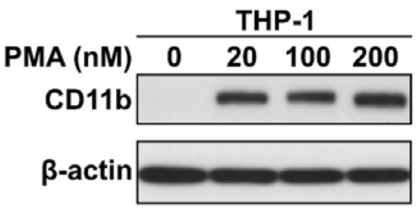

	THP1			
PMA(nM)	0	20	100	200
CD11b	1	217.3585	196.60197	233.6682

Suppl. Fig. 2B

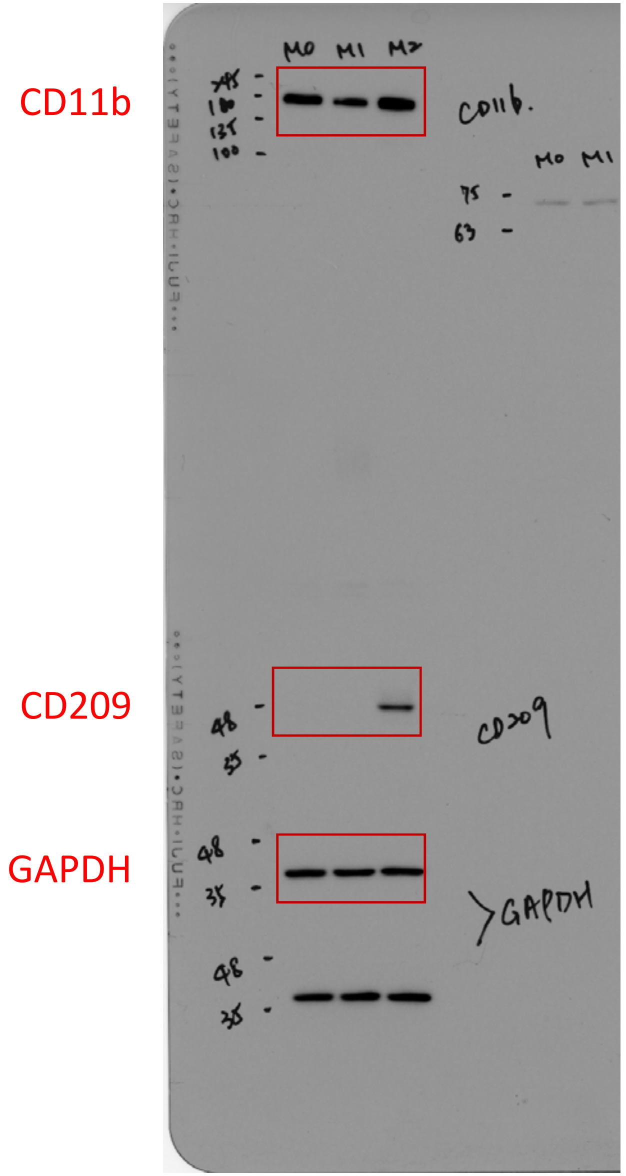

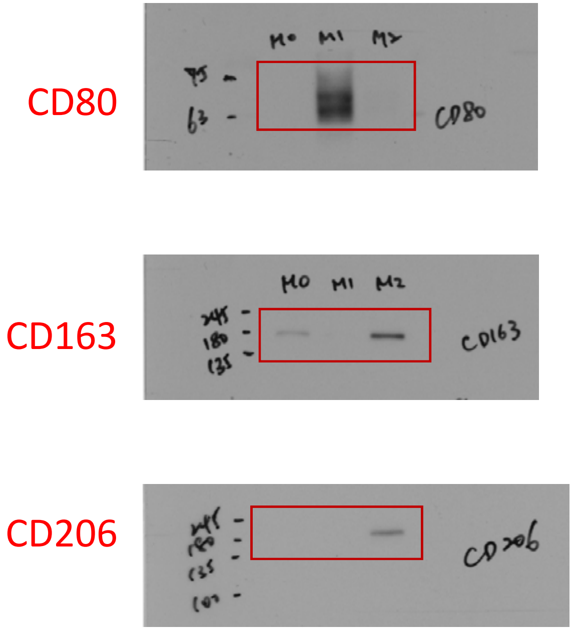

		THP1		
		MO	M1	M2
	CD11b	1.00	0.93	1.02
M1	CD80	1.00	208.79	6.15
	CD163	1.00	0.06	5.41
	CD206	1.00	1.06	64.34
M2	CD209	1.00	3.55	249.32

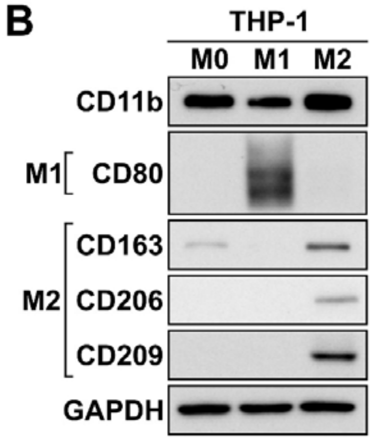

Suppl. Fig. 2D

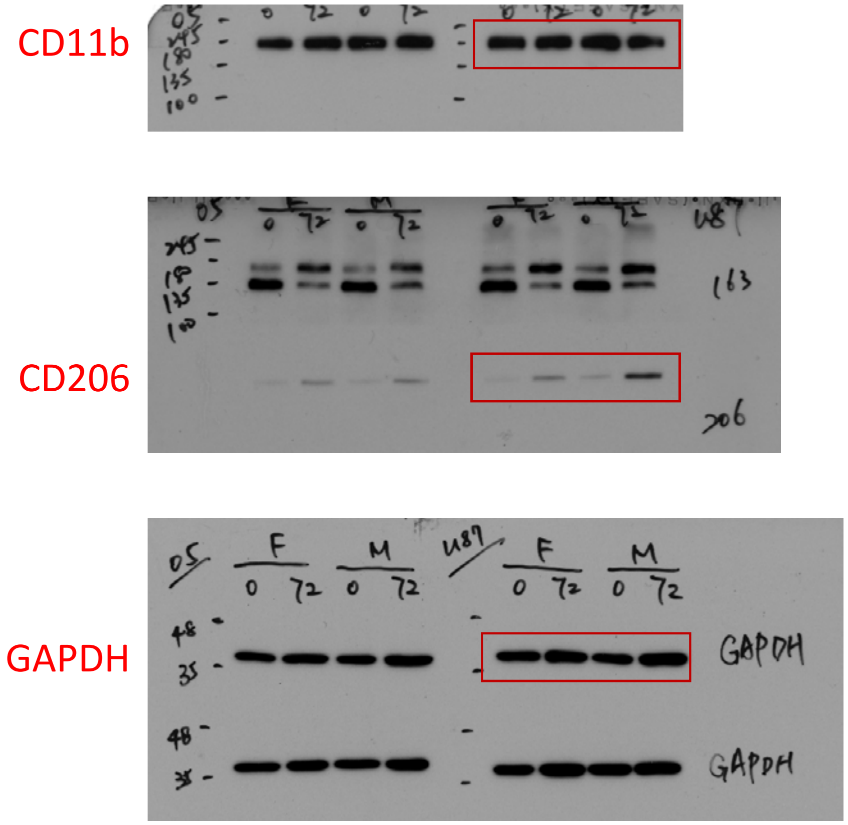

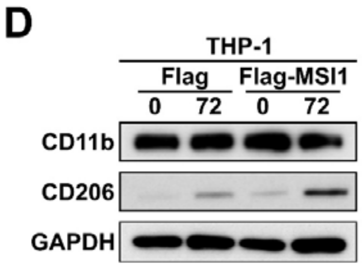

	THP1			
	Flag		Flag-MSI1	
	0	72	0	72
CD11b	1	1.208403	1.243978	0.900839
CD206	1	7.213982	2.751522	20.08676
