# Supplementary material for: Musashi-1 Regulates MIF1-Mediated M2 Macrophage Polarization in Promoting Glioblastoma Progression
Source: Cancers (Basel). 2021 Apr 9;13(8):1799. doi: 10.3390/cancers13081799 (PMC8069545; doi:10.3390/cancers13081799)
Supplement: Supplementary file 1 [file cancers-13-01799-s001.zip › cancers-1125400_supplementary material.pdf]

Supplementary Material

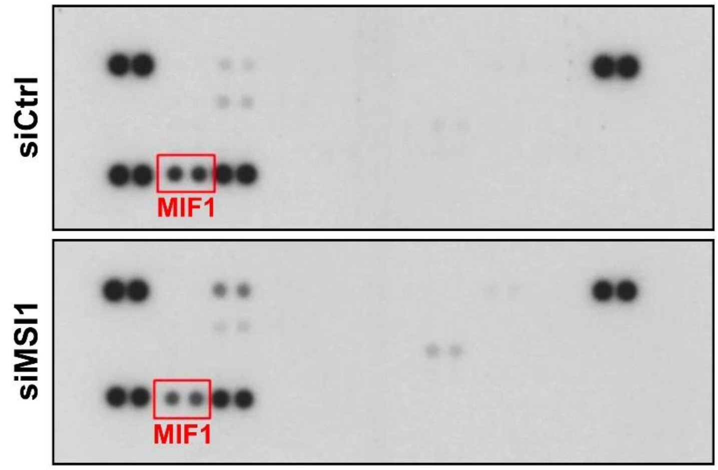

Figure S1. Cytokine array showing decreased secretion of MIF1 by DBTRG-05MG cells upon the knockdown of MSI1.

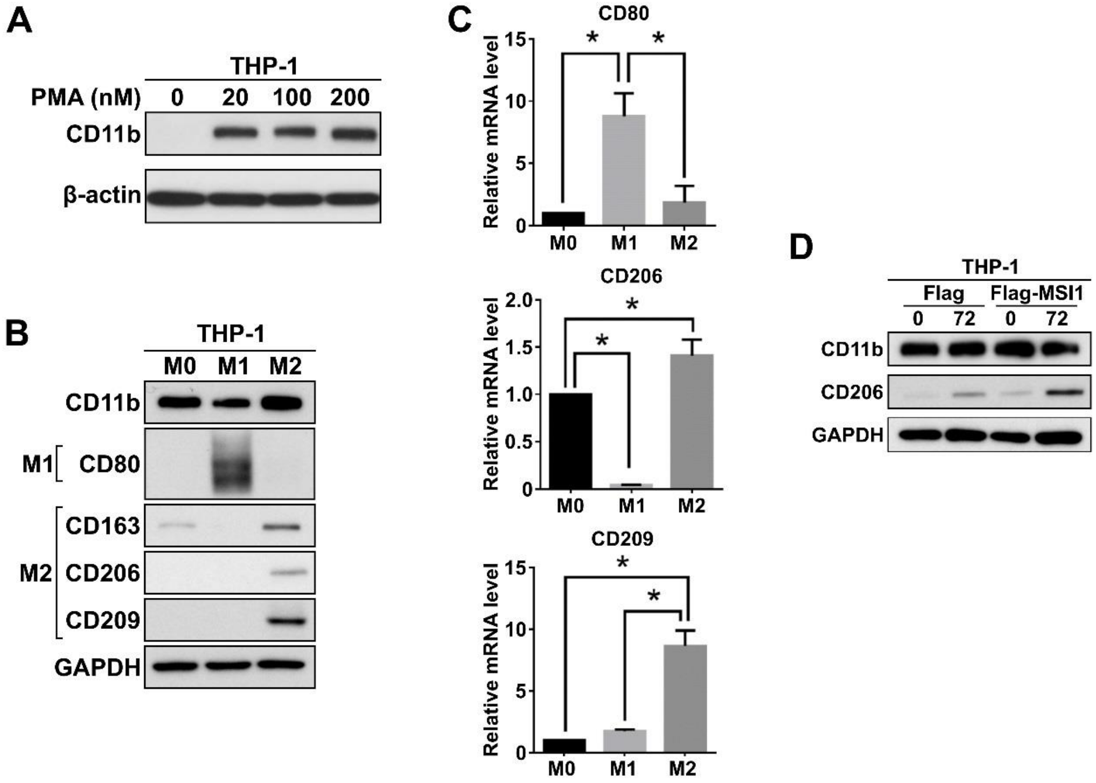

Figure S2. (**A**) Western blot showing the expression of M0 macrophage marker CD11b upon treatment of THP-1 monocytes with the indicated concentrations of PMA. (**B**) The control immunoblot showing the expression of the indicated markers of M1 and M2 macrophage phenotypes in the cells differentiated from M0 state by M1 and M2 standard differentiation protocols, respectively. (**C**) qRT-PCR analysis of the expression of the indicated M1 and M2 markers on the mRNA level in the cells differentiated from M0 state by M1 and M2 standard differentiation protocols, respectively. (**D**) Top panel: immunoblotting analysis of the expression of the indicated CD11b and CD206 in THP-1-derived macrophages treated with the media conditioned by U87MG cells transfected with Flag-MSI1 and control Flag-only plasmid (Flag) for 48 and 72 h. Bottom panel: immunoblotting analysis of the expression of Flag-MSI1 in transfected U87MG cells.

Table S1. Antibodies

**Primary Antibodies**	**Company**	**Product No.**	**Source**
CD11b	Abcam	ab52478	Rabbit
CD80	Abcam	ab134120	Rabbit
CD163	Abcam	ab156769	Mouse
CD206	Abcam	ab64693	Rabbit
DC-SIGN (CD209)	Cell signaling	13193	Rabbit
IL-1β	Abcam	ab9722	Rabbit
Musashi	Cell signaling	85652	Rabbit
Musashi1	Abcam	ab52865	Rabbit
MIF	Abcam	ab65869	Rabbit
Flag	Sigma	F1804	Mouse
GAPDH	Cell signaling	5174	Rabbit
β-Actin	Sigma	5441	Mouse
**Secondary Antibodies**	**Company**	**Product No.**	
Anti-Mouse HRP	Cell signaling	7076	
Anti-Rabbit HRP	Cell signaling	7074	
Alexa Fluor Antibodies	Company	Product No.	Source
DAPI	Sigma	D9542	
Alexa Fluor 555 conjugate	Invitrogen	A-21424	Mouse
Alexa Fluor 488 conjugate	Invitrogen	A-11008	Rabbit
**Flow Cytometry Antibodies**	**Company**	**Product No.**	
Mouse F4/80 PE-conjugated	BioLegend	123109	
Mouse CD80 APC-conjugated	BioLegend	104713	
Mouse CD206 APC-Conjugated	BioLegend	141707	

Table S2. Primer sequences

**Primers used for plasmid construction**		
Name	Sequence (5′-3′)	
MSI1-F	ATGGAGACTGACGCGCCCCAGCCCG	
MSI1-R	TCAGTGGTACCCATTGGTGAAGGCT	
MSI1-F-HindIII	AGAAGCTTATGGAGACTGACGCGCCCCAGC	
MSI1-R-BamHI	AGGATCCTCAGTGGTACCCATTGGTGAAGG	
**PCR primer**		
Name	Forward (5′-3′)	Reverse (5′-3′)
18S	CAGCCACCCGAGATTGAGCA	TAGTAGCGACGGGCGGTGTG
MSI1	ACCGAGGGTTCGGGTTTGTC	GCCGATGCCCAGCATGAAGG
CD80	GCAGGGAACATCACCATCCA	TCACGTGGATAACACCTGAACA
CD206	GGGAAAGGTTACCCTGGTGG	TCAAGGAAGGGTCGGATCGT
CD209	CTAAAGCAGGAGTTCTGGAC	CTAAAGGTCGAAGGATGGAG
IL-1β	AGAAGTACCTGAGCTCGCCA	CTGGAAGGAGCACTTCATCTGT
MIF	CGGACAGGGTCTACATCAACT	TTCTCCCCACCAGAAGGTTG

Tables S3. Chemicals

**Name**	**Company**	**Product No.**
Recombinant Human MIF	PeproTech	300-69
ISO-1	Merck Millipore	478336-92-4
